# Identifying Biomarkers of Frailty Through an Integrated Multi-omics Analysis

**DOI:** 10.1093/geroni/igaf122.232

**Published:** 2025-12-31

**Authors:** John Batsis, Dakota Batchek, David Lynch, Laura Herring, Whitney Stutts, Danae Gross, Hillary Spangler

**Affiliations:** The University of North Carolina at Chapel Hill, Chapel Hill, North Carolina, United States; The University of North Carolina at Chapel Hill, Chapel Hill, North Carolina, United States; University of North Carolina School of Medicine, Chapel Hill, North Carolina, United States; The University of North Carolina at Chapel Hill, Chapel Hill, North Carolina, United States; The University of North Carolina at Chapel Hill, Chapel Hill, North Carolina, United States; The University of North Carolina at Chapel Hill, Chapel Hill, North Carolina, United States; The University of North Carolina at Chapel Hill, Chapel Hill, North Carolina, United States

## Abstract

**Background:**

Older adults are vulnerable to frailty, leading to adverse outcomes. We examined baseline metabolomic/proteomic biomarkers associated with different frailty states with a goal of advancing precision medicine in a pilot study of older adults pursuing physical therapy.

**Methods:**

We conducted a prospective study of 18 adults (≥65 years) from primary care, referred for physical therapy. Frailty was classified using a modified Cumulative Deficit Index (38 variables). Baseline gait speed, grip strength and instrumental activities of daily living (ADL) score (0-100) were assessed. Plasma samples underwent untargeted metabolomics and proteomics. Proteomics used Mag-Net enrichment to detect low-abundance proteins, followed by LC-MS/MS analysis on a Thermo Neo-Orbitrap Astral. Metabolomics involved methanol extraction and CE-MS/MS analysis using a 908Devices ZipChip-Thermo Fusion Lumos.

**Results:**

The mean participant age was 77.1±5.5 (33% female). There were 12 were pre-frail and 6 frail participants. Baseline function differed between groups: gait speed was 1.23±0.26 vs. 0.98±0.21 m/s (p = 0.01), grip strength was 28.8±9.5 vs. 27.1±5.6kg (p = 0.98), and instrumental ADL scores were 100±0 vs. 66.1±22.9 (p = 0.005). Among 4,300 quantified proteins, ∼60 differed (p < 0.05; log2-fold change ±0.5) with ∼40 proteins elevated in frail individuals, including nucleosome-related proteins (H2B, H3C1, H2AC3, H3-4). Metabolomic analysis assessed ∼275 metabolites quantifying 115. Nicotinamide was significantly downregulated in the frail group (p < 0.05; log2-fold <-0.5).

**Conclusions:**

This pilot study suggests that circulating nucleosomes–complexes of histones and DNA–may serve as biomarkers for aging and related conditions. Additionally, nicotinamide, a precursor of NAD^4^, a key coenzyme in metabolism, DNA repair, and mitochondrial function, declines with age, potentially contributing to age-related conditions.

